# Emerging 2D Materials and Their Van Der Waals Heterostructures

**DOI:** 10.3390/nano10030579

**Published:** 2020-03-22

**Authors:** Antonio Di Bartolomeo

**Affiliations:** Physics Department “E.R.Caianiello” and “Interdepartmental center NANOMATES”, University of Salerno, Fisciano, 84084 Salerno, Italy; adibartolomeo@unisa.it; Tel.: +39-089-96-9189

**Keywords:** graphene, MXene, transition metal dichalcogenide, van der Waals heterostructure, heterojunction, photodetection, photovoltaics, water splitting, photocatalysis

## Abstract

Two-dimensional (2D) materials and their van der Waals heterojunctions offer the opportunity to combine layers with different properties as the building blocks to engineer new functional materials for high-performance devices, sensors, and water-splitting photocatalysts. A tremendous amount of work has been done thus far to isolate or synthesize new 2D materials as well as to form new heterostructures and investigate their chemical and physical properties. This article collection covers state-of-the-art experimental, numerical, and theoretical research on 2D materials and on their van der Waals heterojunctions for applications in electronics, optoelectronics, and energy generation.

## 1. Introduction

The advent of graphene [1,2,3], and more recently of two-dimensional (2D) layered materials [4], has opened new perspectives in electronics, optoelectronics, energy generation, and sensing applications [5]. Two-dimensional materials can be fabricated with relatively inexpensive production methods, integrated into existing semiconductor technologies, and offer new physical and chemical properties [6,7,8]. Electrically, they can behave as insulators, semiconductors, metals, or even superconductors. Layered materials consist of covalently bonded and dangling-bond-free layers that can be stuck on top of each other by van der Waals forces to form bulk structures. In general, the number of layers can be controlled to tailor specific properties [9,10]. The possibility to accurately predict the physical properties of layered materials with the exact number of layers is a unique opportunity for directing the design and the fabrication of new electronic and optoelectronic devices.

Different types of 2D materials can form heterojunctions with each other or with bulk materials, without the need for close lattice matching [11]. In these heterojunctions, the weak van der Waals forces between the participant materials do not introduce significant changes at the atomic scale and usually maintain the original electronic structure of the materials. Hence, van der Waals heterojunctions offer the opportunity to combine layers with different properties as the building blocks to engineer new functional materials for high-performance electronic devices, chemical sensors, or water-splitting photocatalysts. A great advantage is that the easy stacking of a variety of 2D materials allows a far greater number of combinations than any traditional growth method [12].

Tremendous amount of work has been done thus far on the physical and chemical properties as well as on the synthesis and the characterization of 2D materials such as graphene [13,14], transition metal chalcogenides [15,16] and dichalcogenides [17,18,19], hexagonal boron nitride [20], black phosphorus [21], organic perovskites [22], etc. Many of these materials have been used to fabricate stacked 2D–2D heterostructures [23], 2D-3D heterojunctions with common bulk semiconductors [24,25], or even 0D–2D and 1D–2D hybrids [26]. The underlying physics and the possible applications in photodetection, biochemical sensing, strain gauges, photovoltaic energy generation, and photocatalytic water splitting have attracted the attention of both theorists and experimentalists. 

This article collection, a reprint of the Special Issue “2D Materials and Van der Waals Heterostructures: Physics and Applications” published by *Nanomaterials* (MDPI), covers state-of-the-art experimental, simulation, and theoretical research on 2D materials and on their van der Waals heterojunctions for applications in electronics, optoelectronics, energy generation, and photocatalysis.

## 2. Emerging 2D Materials and Their Heterostructures

From a material standpoint, the articles of this collection can be organized in three main categories: Graphene and graphene oxide, MXenes and transition metal (di)chalcogenides, and graphene-like materials.

### 2.1. Graphene and Graphene Oxide

First principle calculations are applied to study the electronic and magnetic properties of Stone–Wales defected graphene [27] and the optical properties of graphene/MoS_2_ heterostructures [28], while experimental work is carried out to investigate the properties of graphene/Si Schottky junctions [29] and to realize visible-light driven photoanodes for water oxidation [30]. 

Stone–Wales, formed by the rotation of a C-C bond in a hexagon ring, are the most common topological defects in graphene. They have been widely studied because they can lead to the opening of a band gap that is highly desirable for the application of graphene in electronic devices [31]. Stone-Wales defected graphene (SWG) includes two pairs of pentagonal–heptagonal rings and absorbs molecules more easily than perfect graphene, a feature important for sensor applications. The paper by Xie and coworkers [27] shows that it is possible to tune the band structure of SWG through the interaction with cyclopentadienyl and half-metallocene of Fe, Co, or Ni. The introduction of cyclopentadienyl and half-metallocene increases the conductivity SWG and induces magnetic properties, contributed by the 3D orbital of Fe, Co, Ni, and the molecular orbital of cyclopentadienyl. The study shows that the density of states and the magnetic properties in SWG can be tuned by controlling the cyclopentadienyl and half-metallocene absorption sites.

First principle calculations are also applied by Qiu et al. [28] to demonstrate that the electronic structure and the optical properties of graphene and monolayer molybdenum disulfide (MoS_2_) are changed after they are combined in an heterostructure. MoS_2_ [32,33] is the best known material of the transition metal dichalcogenide (TMD) family that will be treated next. Qiu et al. show that the optical properties of the graphene/MoS_2_ system are improved compared to those of the two separate single-layers. The band gap and the dielectric constants become larger for the graphene/MoS_2_ heterostructure, with redshift for the absorption coefficient, the refractive index, and the reflectance, and blueshift for the energy loss spectrum. 

The heterojunction formed by graphene with traditional 3D materials, which has been a promising research topic [34,35,36,37], is experimentally investigated by Luongo and coworkers [35]. They fabricate graphene/n-Si junctions by transferring graphene on the flat surfaces of Si nanopillars, etched into a Si substrate, and obtain devices with rectifying behavior, remarkable photo-response, and photovoltaic capability. As is typical of good quality graphene/Si junctions [38], their devices exhibit a strongly bias- and temperature-dependent reverse current. Indeed, they report an exponentially growing reverse current below room temperature, which is explained as Schottky barrier lowering caused by the pillar-enhanced electric field, and a quasi-saturated reverse current at higher temperatures, attributed to the dominant effect of carrier thermal generation. 

Graphene oxide is the material produced by oxidation of graphite that includes oxygen functional groups. It has interesting properties that are different than those of graphene. The reduction of the oxygen content through chemical, thermal, and other methods leads to the so-called reduced graphene oxide (r-GO), a cheaper and lower quality form of graphene, yet with important applications [39,40]. r-GO is used by Shuang et al. [30] for the fabrication of photoanodes for water splitting. Sustainable and convenient methods to prepare hydrogen fuel are a worldwide goal. Water splitting through the exploitation of solar energy is the most appealing method to obtain hydrogen fuel. The standard photocatalytic process for water splitting can be facilitated by the application of an external potential in the so-called photoelectrochemical approach. For this reason, many research efforts have been addressed to the fabrication of semiconducting nanomaterials with enhanced photoelectrochemical properties under visible light and, in particular, to the development of materials for photoanodes. Shuang et al. [30] propose a novel composite material consisting of β-Cu_2_V_2_O_7_ nanoparticles deposited on TiO_2_ nanorods followed by the addition of r-GO flakes. They show that electrophoretic deposition of p-type r-GO flakes on β-Cu_2_V_2_O_7_/TiO_2_ nanorods remarkably improves the durability, charge transfer resistance, and photocurrent density. 

### 2.2. MXenes and Transition Metal (di)chalcogenides

MXenes are a new, large family of layered materials consisting of transition metal carbides, nitrides, and carbonitrides. Examples of mono and double transition metal MXenes are Ti_2_C, Ti_4_N_3_, Ti_3_CN, Mo_2_N, Mo_2_TiC_2_, Mo_2_Ti_2_C_3_, Cr_2_TiC_2_, etc. MXenes such as Nb_2_CT_x_, Ti_2_CT_x_, or Ti_3_C_2_T_x_, with the surface terminated by T_x_ functional groups (e.g., O, F, OH, Cl), combine a high metallic conductivity with the hydrophilic nature of their hydroxyl or oxygen terminated surfaces, behaving as a sort of “conductive clays”. MXenes have been applied to energy storage, water purification, chemical catalysts, electrochemical sensors, field effect transistor sensors, gas sensors, etc. [41]. 

Transition metal (mono)chalcogenides (TMC) [42] are MS, MSe, and MTe compounds (sulfides, selenides, tellurides of transition metals, M, such as titanium, niobium, molybdenum, cadmium, tungsten, etc.) and differ considerably from the transition metal oxides, MO, both in their structures and chemical and physical properties. Compared to oxygen atoms, the S, Se, and Te chalcogen atoms are larger, less electronegative, and have d orbitals (3d for S, 4d for Se, 5d for Te). Consequently, the metal-chalcogen bonds are more covalent than the metal-oxygen bonds and often involve the d orbitals. The increased covalency leads to broad valence and conduction bands with an energy gap generally narrower than in the case of oxides. Typically, the gap in sulfides is 1–3 eV, becomes smaller in selenides, and can vanish in tellurides. The availability of d orbitals in the calchogens causes large polarizability of chalcogenide ions. Furthermore, the extended d orbitals of the chalcogens can mix with metal d orbitals and have a stabilizing effect, causing for instance the smaller solubility of MS, MSe, and MTe compared to MO products in water.

Transition metal dichalcogenides (TMDs) are the most studied 2D materials after graphene [17,43]. They consist of a monolayer of transition metal atoms sandwiched between two layers of chalcogen atoms in a hexagonal or pentagonal lattice. Their standard structural formula is MX_2_, where M represents a transition metal and X denotes the chalcogen. Molybdenum disulfide (MoS_2_) and diselenide (MoSe_2_) [44,45,46,47,48] and tungsten disulfide (WS_2_) and diselenide (WSe_2_) [49,50] are the most common TMDs with a hexagonal structure, while palladium diselenide (PdSe_2_) [10,51,52] is the prototype of TMDs with a pentagonal structure. Peculiarities such as absence of a dangling bond, electrocatalytic properties, chemical stability, mechanical flexibility, strong coupling to light, and bandgap tunable by number of layers and ranging from semi-metallic to over 2 eV, make TMDs materials of choice for the development of new sensors, electronic devices, and water-splitting photocatalysts. Indeed, different types of TMD-based biological sensors [53], flexible gas sensors [54], 3d image sensors [55], and tactile sensors [56], as well as large-scale electronic devices and circuits including logic, memory, optoelectronic, and analog devices [57] or photocatalysts for use in pollutant degradation and hydrogen evolution [58], have been demonstrated.

Xu et al. [59] propose Ti_3_C_2_T_x_ MXene in combination with transition metal dichalcogenides to design new optical sensors based on surface plasmon resonance (SPR) to use for biosensing and chemical sensing. Highly sensitive SPR sensors have been previously demonstrated with coating of dielectric materials, graphene, or TMDs on metal films [59,60]. The new prism-coupled SPR sensor is based on Au-Ti_3_C_2_T_x_-Au-TMDs in a modified Kretschmann configuration. The theoretical works of the authors show that it possesses enhanced sensitivity as compared to the bare Au film-based SPR sensor.

The synthesis of large area and good quality TMDs is of paramount importance for their industrial exploitation. The fabrication of monolayer WS_2_ flakes is the subject of the work of Shi and coworkers [61]. WS_2_ has a structure similar to the best-known MoS_2_, but exhibits stronger photoluminescence quantum yield at room temperature and larger spin-orbit coupling and is a promising 2D material for applications in optoelectronics and spintronics. The difficulty of controlling the interrelated growth parameters makes the synthesis of large-area monolayer WS_2_ still challenging. Therefore, the reported one-step chemical vapor deposition (CVD) process, by direct sulfurization of powdered tungsten trioxide (WO_3_) drop-casted on SiO_2_/Si substrates at atmospheric pressure that yields large-area monolayer WS_2_, is of great interest. Triangular monolayer WS_2_ flakes with edge lengths up few hundred microns and homogeneous crystallinity are obtained.

CVD monolayer WSe_2_, which is a TMD structurally similar to MoS_2_, but with a slightly lower bandgap (1.6 eV), is used in back-gated field effect transistors (FETs) by Urban et al. [62]. Two-dimensional WSe_2_ exhibits strong optical absorption in the visible range, a good light-to-electricity conversion coefficient, and forms FETs with ambipolar behavior with most of the metals commonly used in electronics. Urban and coworkers fabricate WSe_2_ backgate FETs with Ni Schottky contacts and measure their electrical characteristics under different environmental conditions. They demonstrate that lowering the pressure in air-exposed WSe_2_ dramatically affects the electrical characteristics turning the FET conduction from the p- to n-type. Furthermore, from the electrical characterization at different temperatures, a gate modulation of the Schottky barrier (SB) at the contacts was proven. In addition, the work reports the temperature dependence of the carrier mobility and the subthreshold swing, as well as the photo response at several laser wavelengths. Some of the results are likely qualitatively valid for other TMD-based devices.

First principle calculations are again performed to investigate the electronic and optical properties of the β-polytypes of indium selenide, β-InSe [63], and carbon selenide, β-CSe [64], as well as to study the properties of Pd_2_Se_3_ [65]. Recently, much attention has been placed on the first two materials which belong to the family of layered TMC [66]. 

β-InSe is the most stable phase of InSe, due to the ABAB crystal stacking mode. The individual layer has a hexagonal structure and shows different electronic and optical properties compared to the bulk [40]. Monolayer and few-layer β-InSe possess moderate band gaps of 2.4 eV and 1.4 eV, respectively, and can be optimal candidates for use in broadband optoelectronic devices. Moreover, the appreciable shift of the valence band maximum upon thickness variation is very important for optimizing the band gap and improving the mobility of electrons and holes. The study presented by Sang et al. [63] shows that the electronic band structure, the work function, and optical properties of β-InSe are strongly dependent on the number of layers. For instance, the band structure exhibits direct-to-indirect transition from bulk β-InSe to few-layer β-InSe and the work functions varies in the 4.77–5.22 eV range depending on the number of layers. Similarly, the thickness variation strongly affects the imaginary part of the dielectric function which determines the optical properties of the material. 

Based on numerical work, Zhang at al. propose carbon selenide, β-CSe, as a novel ultra-thin stable material with wide indirect bandgap. The β-CSe exhibits slightly anisotropic mechanical characteristics and its bandgap and band-edge curvature are sensitive to in-plane strain, causing the carrier effective mass to be strain-dependent. Zhang and coworkers also propose a heterojunction obtained by stacking α-CSe on a β-CSe sheet. The α-CSe/β-CSe interface constitutes a type-II van der Waals p-n heterojunction with strong built-in electric field across the interface, due to the charges transferring from β-CSe to α-CSe. The built-in potential causes substantial energy band bending, which can be exploited to spatially separate photo-generated carriers in photovoltaic devices. Furthermore, as a metal-free photocatalyst, the α-CSe/β-CSe heterojunction is endowed with an enhanced solar-driven redox ability for photocatalytic water splitting via reduced electron-hole-pair recombination. This study will likely motivate experimental research on the synthesis and the properties of this new material as well as on the design of new devices.

Layered materials based on noble transition metals, such as Pd and Pt, have gained popularity after the successful exfoliation of PdSe_2_ [51,67,68] and the discovery of its stability in air. Following the observation of the formation of Pd_2_Se_3_ by interlayer fusion of PdSe_2_ [69,70], Li and coworkers [65] study the physical properties and device applications of monolayer Pd_2_Se_3_ by first principles calculations. They demonstrate that Pd_2_Se_3_ monolayers have a great potential as absorber material in ultrathin photovoltaic devices owing to their quasi-direct band gap of 1.39 eV, high electron mobility (> 100 cm^2^V^−1^s^−1^), and strong optical absorption (~10^5^ cm^−1^) in the visible solar spectrum. Furthermore, the Pd_2_Se_3_ bandgap can be modulated and changed from indirect to direct by biaxial strain. More importantly, monolayer Pd_2_S_3_, obtained by replacing Se with S, is stable and can be used in a vertical stack with Pd_2_Se_3_ monolayer to form a type-II heterostructure. The simulation indicates that such a structure, used as a solar cell, could achieve 20% power conversion efficiency, higher than that of MoS_2_/MoSe_2_ photovoltaic devices. This numerical study presents several challenges for new experiments on 2D Pd_2_Se_3_ and Pd_2_S_3_ as well as on the application of their heterostructures for efficient solar energy conversion.

### 2.3. Graphene-like Materials

The quest for efficient water-splitting photocatalysts motivates the first principle studies by Wang and coworkers [71,72], in which graphene-like carbon nitride, g-C_3_N_4_, and zinc oxide, g-ZnO, are used to form 2D heterojunctions with transition metal (di)chalcogenides. The investigation of CdS/g-C_3_N_4_ [71] and g-ZnO/WS_2_ [72] heterostructures aims to find appropriate strategies to modulate the electronic and photocatalytic properties of the individual materials. Indeed, both cadmium sulfide CdS [73] and graphitic carbon nitride g-C_3_N_4_ [74] have suitable bandgaps for visible light absorption (2.4 and 2.7 eV, respectively) and could be good photocatalysts, but the former lacks stability due to the self-oxidation of photogenerated species while the latter has poor photocatalytic efficiency because of the fast recombination of photogenerated electron–hole pairs. However, significantly improved photocatalytic activity, as compared to the individual 2D materials, can be achieved with their heterostructure. The built-in electric field, due to the charge accumulation/depletion around the interfaces, promotes the effective separation and migration of photogenerated carriers, which is beneficial for the photocatalytic performance. Hence, Wang et al. investigate the energetic, electronic and optical properties, and the band edge alignments of CdS/g-C_3_N_4_ as a function of biaxial strain and pH of the electrolyte, with the objective of optimizing the photocatalytic performances. The numerical simulations show that monolayer CdS weakly contacts with g-C_3_N_4_, forming a type II van der Waals (vdW) heterostructure. The predicted bandgaps and optical absorptions indicate that such a heterostructure can absorb visible light and the induced built-in electric field at the interface promotes the effective separation of the photogenerated carriers. Furthermore, the interface adhesion energy, bandgap, and band edge positions can be adjusted by applying a biaxial strain.

The water-splitting photocatalytic properties of the heterostructures formed by g-ZnO with two-dimensional WS_2_ or WSe_2_ are studied as a function of the rotation angles and the biaxial strain [72] as well. g-ZnO has been experimentally synthesized [75] and density functional theory studies have suggested that doping with nonmetal species can endow it with tunable magnetism [76]. Wang and coworkers demonstrate that g-ZnO/WS_2_ heterostructures with appropriate rotation angles possess a suitable bandgap for visible absorption, proper band edge alignment, and effective separation of carriers, being promising visible water-splitting photocatalysts. Conversely, the water oxygen process of the ZnO/WSe_2_ heterostructures is limited by their band edge positions.

These theoretical findings offer a sound basis to develop CdS/g-C_3_N_4_ or g-ZnO/WS_2_-based water-splitting solutions to prepare hydrogen fuel.

## 3. Conclusions

Although graphene has proven to be an unmatched material due to its abundance of properties and applications, other 2D materials have been isolated or synthesized to better serve specific needs. Owing to their bandgaps, an example of a feature that graphene does not possess, transition metal (di)chalcogenides have emerged for instance as materials well-suited for electronics, optoelectronic, and photocatalytic applications.

The layer control in two-dimensional materials provides an effective strategy to modulate their optical and electrical properties. The formation of van der Waals heterostructures, with enhanced features, offers a unique opportunity to fabricate new materials.

This book offers a collection of research papers that cover the state-of-the-art of fabrication, properties, and applications of graphene and graphene-like materials, along with their heterostructures.

The electronic and optical properties of several popular 2D materials and their heterojunctions, as well as the opportunities for their exploitation in sensors, electronic and optoelectronic devices, and water-splitting photocatalysts, are extensively discussed. New 2D materials and new heterostructures like α-CSe/β-CSe, Pd_2_Se_3_/Pd_2_S_3_ or CdS/g-C_3_N_4_, and g-ZnO/WS_2_ are proposed based on first principle calculations.

These studies consolidate and extend the general knowledge of the physics and technology of 2D materials and offer many theoretical results that will likely be the challenging subject of forthcoming experimental investigations.
